# Hyperemia-Related Changes in Arterial Stiffness: Comparison between Pulse Wave Velocity and Stiffness Index in the Vascular Reactivity Assessment

**DOI:** 10.1155/2012/490742

**Published:** 2012-08-07

**Authors:** Juan Torrado, Daniel Bia, Yanina Zócalo, Ignacio Farro, Federico Farro, Ricardo L. Armentano

**Affiliations:** ^1^Physiology Department, School of Medicine, Centro Universitario de Investigación, Innovación y Diagnóstico Arterial (CUiiDARTE), Republic University, General Flores 2125, 11800 Montevideo, Uruguay; ^2^Faculty of Engineering and Natural and Exact Sciences, Favaloro University, Belgrano 1723, C1093AAF Buenos Aires, Argentina

## Abstract

Carotid-to-radial pulse wave velocity (PWV_cr_) has been proposed to evaluate endothelial function. However, the measurement of PWV_cr_ is not without limitations. A new simple approach could have wide application. *Stiffness index* (SI) is obtained by analysis of the peripheral pulse wave and gives reproducible information about stiffness of large arteries. This study assessed the effects of hyperemia on SI and compared it with PWV_cr_ in 14 healthy subjects. Both were measured at rest and during 8 minutes after ischemia. SI temporal course was determined. At 1 minute, SI and PWV_cr_ decreased (5.58 ± 0.24 to 5.34 ± 0.23 m/s, *P* < 0.05; 7.8 ± 1.0 to 7.2 ± 0.9 m/s; *P* < 0.05, resp.). SI was positively related to PWV_cr_ in baseline (*r* = 0.62 , *P* < 0.05), at 1 minute (*r* = 0.79, *P* < 0.05), and during the whole experimental session (*r* = 0.52, *P* < 0.05). *Conclusion*. Hyperemia significantly decreases SI in healthy subjects. SI was related to PWV_cr_ and could be used to facilitate the evaluation of hyperemia-related changes in arterial stiffness.

## 1. Introduction

Normally, endothelial cells release the powerful antiatherogenic and smooth muscle relaxing factor, that is, nitric oxide (NO), in response to a rise in blood flow [1]. “Endothelial dysfunction” can be understood as a loss in this capability and is considered a main step in the ignition and progression of atherosclerosis, a major cause of cardiovascular (CV) events (mainly stroke and myocardial infarction) [2, 3]. Celermajer's et al. technique has become the most popular test to assess endothelial function (EF) [4], which consists in positioning a pneumatic cuff around the upper arm and to determine an arterial occlusion for five minutes (transient ischemia). This maneuver elicits an increment of blood flow in the brachial artery when the cuff is deflated (i.e., reactive hyperemia, RH). RH finally stimulates endothelium to release NO [5], resulting in healthy subjects in a dilatation of the brachial artery and wall intrinsic alterations [6].

Evaluation of EF has demonstrated clinical importance in terms of cardiovascular (CV) risk assessment, positioning as an independent risk factor of CV events and improving the classification of subjects as low, intermediate, and high CV risk compared with the employment of assessing traditional risk factors alone [3]. Although evaluation of EF could have a defined place in the clinical practice, technical difficulties of available techniques for the assessment EF have been reported [7–9].

In the past five years, RH-related changes in pulse wave velocity (PWV) have been proposed as a potential tool to evaluate EF [6, 9]. Carotid-to-femoral PWV is recognized as the “gold standard” parameter for the evaluation of regional arterial stiffness and has had a wide biomedical application [10, 11]. Previous studies have shown that PWV can be acutely altered by endothelium-related changes in vascular tone and constitutively released NO [12, 13]. About this, Kinlay et al. demonstrated that PWV decreased and augmented in response to nitroglycerin (NO-donor) and L-NMMA (NO synthase inhibitor) administration, respectively [13]. Taking into account the link between endothelial factors and arterial stiffness regulation, changes in arterial stiffness in response to RH (most common endothelial stimulus) have been proposed for the evaluation of EF. Previously, it has been reported a reduction in carotid-to-radial PWV (PWV_cr_) values in response to RH test in healthy young adults and a blunted or low reduction in pathophysiological circumstances [6, 9, 14–17] Potential clinical value of assessing PWV_cr_ was only reported in the context of vascular reactivity assessment but it has not been found useful in predicting cardiovascular events in baseline conditions, as has carotid-to-femoral PWV [10].

The analysis of the peripheral pulse wave (pulse wave analysis, PWA) can provide parameters with different meanings in the arterial dynamics, some already used to assess the EF and arterial stiffness [18–20]. *Stiffness index *(SI) a PWA derived parameter developed by Millasseau et al., normally obtained from the analysis of the contour of the digital volume, gives reproducible information about arterial stiffness (the higher the stiffness, the higher the SI values) [21]. It has been demonstrated previously that the contour of the digital volume pulse contains similar information to that of peripheral pressure pulse [22]. Analyzing the EF with simple and inexpensive parameter, such SI is recognized, would facilitate the EF assessment, since only a mechano-transducer placed over the radial artery would be necessary. However, it is unknown if the RH-related changes in arterial dynamics can be assessed by SI obtained from the pulse waveform (mechanotransducers) by means of PWA.

In this context, this work's aim was to determine and characterize the SI and PWV_cr_ temporal profile in response to transient ischemia in the forearm. In addition, the relationship between SI and PWV_cr_ in stable conditions and during RH was analyzed.

## 2. Methods

### 2.1. Subjects

Healthy and untrained medicine students (*n* = 14) randomly selected were invited and agreed to participate in the study. Following the guidelines for ultrasonic assessment of brachial FMD, subjects were asked to abstain from physical activity, tobacco products, and vitamin supplementation for at least 6 hours prior to the examination [23]. The study protocol was approved by the Institutional Ethic Committee (Republic University). Informed consent was obtained. 

The subjects' main characteristics are detailed in Table 1.

### 2.2. Study Protocol and Recordings

Subjects' height and weight were measured, and the body mass index (BMI, weight to height squared ratio) was calculated. Venous blood samples were drawn and processed immediately to obtain laboratory data (Table 1). 

**Figure 1:**
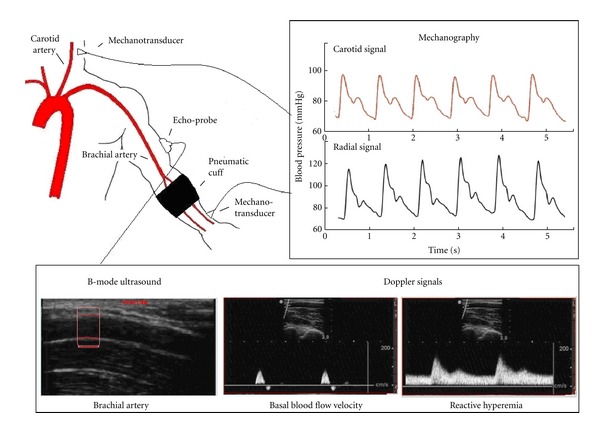
Schema of the instrumental approach employed to measure the PWV_cr_ and SI (mechanotransducers), brachial artery diameter (B-mode ultrasound), and blood flow velocity (Doppler signals). Note the carotid and radial pulse pressure (mechanography), the longitudinal view of the brachial artery (B-mode ultrasound), and the brachial blood flow velocity (Doppler signals) before and after the cuffocclusion and deflation were performed. Immediately after the cuff deflation, there is an acute and transient increase in brachial blood flow velocity (RH).

During the second part of the experimental session, subjects were instructed to lie in supine position for 15 minutes to establish a hemodynamic steady state in a temperature-controlled room (21°–23°C). Heart rate (HR) and right brachial blood pressure (BP) were measured using an oscillometric device (Omron HEM-433INT Oscillometric System; Omron Healthcare Inc., Illinois, USA) every two minutes during the whole study. HR was also determined from the analysis of the carotid and radial signals obtained by mechanotransducers (see below). Upper limb transient ischemia (five minutes) was caused by a pneumatic cuff placed in the left forearm and inflated to approximately 50 mm Hg above the systolic pressure (Figure 1) [23].

Before (baseline) and during 8 minutes after cuff deflation, carotid and radial pressure waveforms were simultaneously obtained using strain gauge mechanotransducers (Motorola MPX 2050, Motorola Inc., Corporate 1303 E. Algonquin Road, Schaumburg, IL 60196, USA) placed on the skin over the carotid and radial arteries. Signals were recorded and analyzed off-line using software (developed by our group) that allows obtaining SI and PWV_cr_ [14]. SI was calculated taking into account the subject height and the time delay between the systolic and diastolic peaks (Δ*t*_*S*-*D*_) of the radial pressure wave [21]:

(1) $$\begin{array}{c} \text{SI}=\frac{\text{height}}{{\Delta t}_{S\text{-}D}}. \end{array}$$

PWV_cr_ was obtained considering the given distance between the measurement sites (Δ*x*) and the time delay (Δ*t*_*C*-*R*_) between the carotid and radial waves onset:

(2) $$\begin{array}{c} {\text{PWV}}_{\text{cr}}=\;\frac{\Delta x}{{\Delta t}_{C\text{-}R}}. \end{array}$$

The algorithm used to detect the waves foot is explained in previous work [14].

At the same time, left brachial artery was visualized longitudinally above the antecubital crease using high resolution B-Mode ultrasound (SonoSite, MicroMaxx, SonoSite Inc., 21919 30th Drive SE, Bothell, WA 98021, USA) (brachial artery diameter obtainment) and Doppler signals were performed in order to determine blood flow velocity in baseline and during postischemia for the characterization of endothelial stimulus. The latter was analyzed by means of shear rate, an estimate of shear stress without accounting for blood viscosity [24]. For this purpose, the mean blood flow velocity (*Vm*) and brachial diameter (*D*) were related as follows:

(3) $$\begin{array}{c} \text{SR}=\frac{Vm}{D}. \end{array}$$

All measurements were done by the same trained operator. Measurement variation coefficient was less than 5%. 

**Figure 2:**
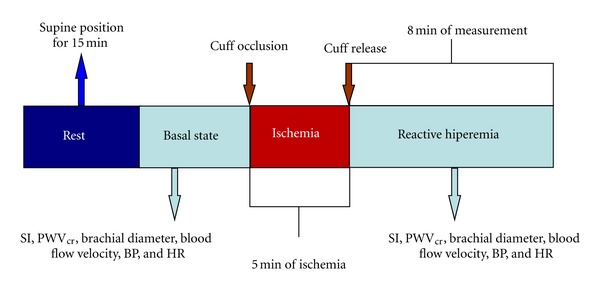
Representative diagram of the study protocol applied to evaluate changes in arterial biomechanics by means of PWA (stiffness index) and PWV.

The study protocol is represented in Figure 2.

### 2.3. Data Analysis

SI and PWV_cr_ were determined at baseline and during 8 minutes after cuff deflation. The changes in SI and PWV_cr_ with respect to basal conditions were quantified as

(4) SI  [%]  =  SIafter  cuff-deflation−SIbaselineSIbaseline·100,PWVcr[%]=PWVcr  after  cuff-deflation−PWVcr  baselinePWVcr  baseline·100.

### 2.4. Statistical Analysis

Data are shown as mean values (MV) ± standard deviation (SD). Results in figures are presented as MV ± standard error of the mean (SEM). Changes in BP, HR, SI, PWV_cr_, arterial diameter, and shear rate were evaluated using ANOVA + Bonferroni test. Changes in the studied parameters (PWV_cr_, SI, arterial diameter, and shear rate percentage changes) were evaluated using two-tailed paired Student *t*-test. Linear regression analysis was used to assess the relationship between the SI and PWV_cr_. A *P* < 0.05 was considered significant.

## 3. Results

All subjects were included in the analysis. No hypertensive levels of blood pressure or abnormal values in the serum parameters were found in the subjects (Table 1). There were no significant changes in HR or brachial BP during the studies. Shear rate values were higher and maximal with respect to baseline immediately when the cuff was deflated (46 ± 24 to 180 ± 79 s^−1^; *P* < 0.05). 

**Figure 3:**
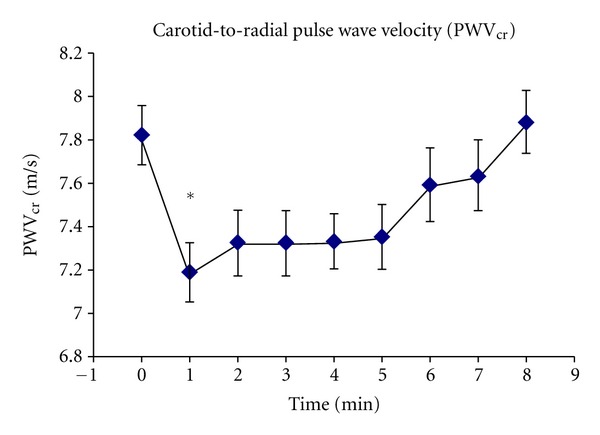
MV and SEM of PWV_cr_ temporal pattern at rest (baseline) and 8 minutes after cuff deflation. *indicates significance comparing baseline to one minute after release of the cuff.

As it can be seen in Figure 3, the maximal mean reduction (8.0%) in PWV_cr_ was reached at one minute after cuff release (7.8 ± 1.0 to 7.2 ± 0.9 m/s; *P* < 0.05). A recovery trend in PWV_cr_ was observed thereafter and eight minutes after-cuff-release basal levels were recovered. 

**Figure 4:**
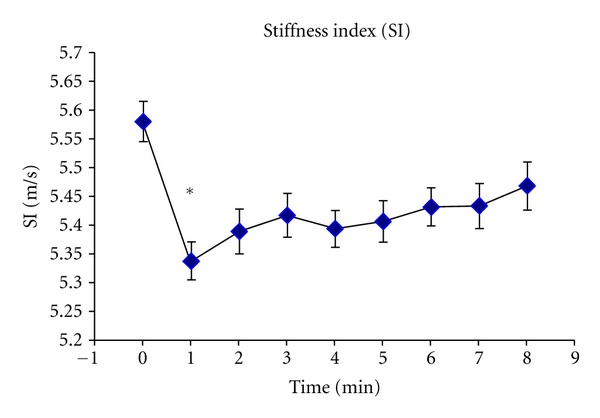
MV and SEM of SI temporal pattern measured at rest (baseline) and 8 minutes after cuff deflation. *indicates significance (*P* < 0.05) comparing baseline to one minute after cuff deflation.

**Figure 5:**
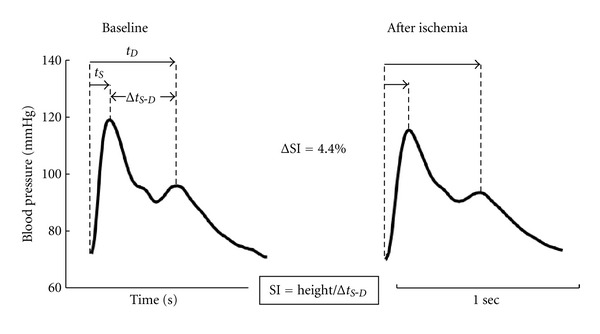
An example of the effect of forearm ischemia and RH on the radial pressure waveform in a single individual. The changes in the wave shape are quantified using the stiffness index (SI), calculated as the relationship between the subject height and the time delay in the systolic and diastolic peaks (Δ*t*_*S*-*D*_). It can be seen that Δ*t*_*S*-*D*_ is increased (reducing SI value) by the 5 minutes of occlusion and cuff deflation. *t*_*S*_ and *t*_*D*_ are time from foot to peaks systolic and diastolic, respectively.

Figure 4 shows basal SI levels and its temporal profile after cuff deflation. As can be seen, the maximal SI change (4.3%) was observed one minute after cuff deflation (5.58 ± 0.24 to 5.34 ± 0.23 m/s; *P* < 0.05). Thereafter, SI values showed a recovery trend, without reaching basal levels. Typical pulse wave traces obtained during basal conditions and one minute after cuff deflation are depicted in Figure 5. In Table 2 are shown the delays of the radial pulse pressure waveform. As it can be seen, the major change due to RH/transient ischemia was obtained in the time delay between foot and second (diastolic) peak of the radial pulse. 

**Figure 6:**
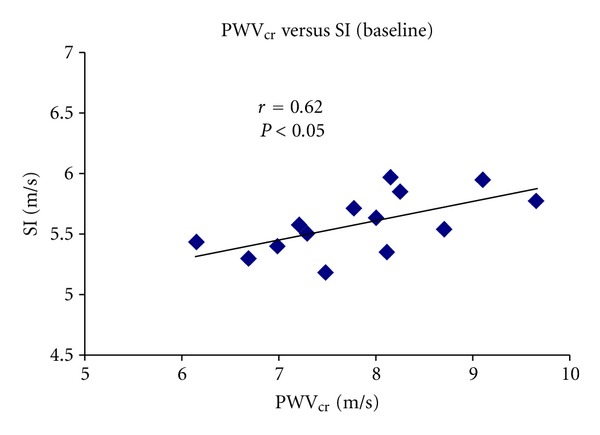
Simple regression analysis comparing SI and PWV_cr_ in basal conditions.

**Figure 7:**
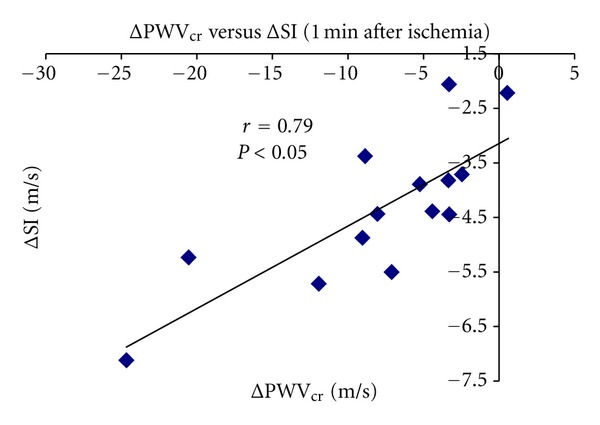
Relationship between ΔSI% and ΔPWV_cr_% one minute after ischemia.

**Figure 8:**
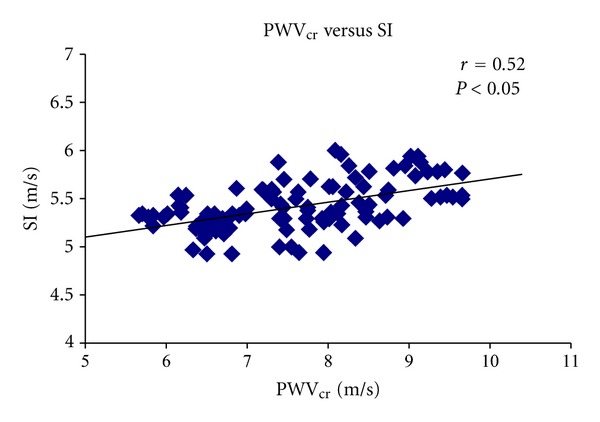
Relationship between SI and PWV_cr_ during the whole experimental session.

SI and PWV_cr_ levels correlated during basal conditions (*r* = 0.62, *P* < 0.05; Figure 6). A higher correlation was obtained in the relationship between SI and PWV_cr_ one minute after the cuff deflation (*r* = 0.79, *P* < 0.05; Figure 7). Finally, SI and PWV_cr_ correlated during the whole experimental session, that is to say during basal condition and the 8 minutes of postischemia (*r* = 0.52, *P* < 0.05; Figure 8). 

## 4. Discussion

Up to now, methods currently available, for example, FMD assessed by ultrasound devices, vary considerably across populations. These discrepancies could be due to differences in study populations (and thus cardiovascular risk factor profiles) and/or a consequence of methodological. The latter is of importance when attempting to provide reference values of FMD and to achieve a wide application and diffusion of EF evaluation in clinical practice [7]. 

Recently, it has been proposed to assess EF by means of the analysis of the PWV_cr_ changes after transient ischemia [6]. In healthy adults transient ischemia and the consequent RH result in a PWV_cr_ reduction, indicating a decrease in the arterial stiffness [6, 14–17]. In this work we propose evaluating the arterial stiffness changes associated with RH/transient ischemia using the PWA-derived SI. 

The radial pulse waveform is formed as a result of the interaction between the left ventricle and systemic circulation [20]. It usually exhibits an early systolic peak and a later peak that occurs a short time after the first peak in early diastole (Figure 5). The systolic component of the waveform arises mainly from a forward-going pressure wave transmitted along a direct path from the left ventricle to the radial measurement site. However, the diastolic component arises from pressure waves from probably two different origins: those transmitted along the aorta to small arteries in the lower body, from where they are then reflected back along the aorta as a reflected wave which then travels to the radial artery, and those reflected back from the distal part of the upper arm to radial measurement site [25]. The transit time between the systolic and diastolic peaks(Δ*t*_*S*-*D*_) is influenced by the PWV of pressure waves in the aorta and large arteries, which is proportional to subject height [21]. SI cannot be expected to provide identical information to PWV, since the contour of the peripheral pulse is complex and it is likely to be influenced by factors other than PWV, as wave reflections [21].

After transient ischemia of the forearm we found simultaneously reduction in SI and PWV_cr_, which is consistent with a reduction in arterial stiffness. However, SI maximum changes were smaller than those of the PWV_cr_ (4.3% versus 8.0%) indicating less sensitiveness and/or participation of different mechanisms. One important point to be mentioned, is that the maximal changes found in both SI and PWV_cr_ were evidenced one minute after release of the arterial occlusion, which is consistent with the normal and major dilatation (FMD) with clinical meaning [23, 26]. 

Simple regression analysis showed a significant and positive correlation between PWV_cr_ and SI during the different states (Figures 6–8). However, the smaller reduction of the SI values and the relative strong correlations with PWV_cr_ response could be indicating similarities but differences as well between both parameters. The relationship between ΔPWV_cr_ and ΔSI might be explained only partially on simply physical principles. As PWV is reduced in the vascular segment (i.e., the foot of the wave is delayed) it would take longer for other features of the pulse to reach the peripheral measurement point. We found increases in both delays, between the foot and first peak (systolic) and between the foot and second peak (diastolic) of the radial pulse. However, although the delay from foot to first peak of radial pulse increased probably as a result of a reduction of PWV in the vascular segment, the changes in the delay from the foot to second peak were greater increasing Δ*t*_*S*-*D*_ (Table 2). When the inflated cuff is inducing the transient ischemia added to the rise of blood flow that is evoked after the arterial release, geometrical (i.e., increase of arterial diameter) and intrinsic wall (i.e., arterial stiffness) changes as well as alterations of the amount of wave reflection and also changes in sites of wave reflections in the distal part of the upper arm could explain the similarities and differences observed between PWV_cr_ and SI. SI and PWV_cr_ temporal courses were also slightly different. Whereas PWV_cr_ values reached baseline levels during the study time, SI remains low even 8 min after release of the ischemia, showing only a tendency to recovery without reaching definitely baseline conditions. This difference in SI and PWV could be related to vascular local changes induced by ischemia that maintains SI reduced, rather than changes in regional arterial stiffness. 

In this work, we demonstrated for the first time, that in normal young adults the vascular reactivity tested by the provocation of RH/transient ischemia can be evaluated measuring SI. Through the utilization of RH, a usual endothelial stimulus, this work provides only indirect information about a potential role of EF in SI changes. Then, at least in theory, SI analysis could be used in the clinical practice to evaluate the endothelial dynamics. This could represent an advantageous approach over those nowadays employed in the EF evaluation given its robustly, simplicity, operator independence, and relative low costs of required devices. Future works are needed to elucidate the exact mechanisms involved in SI behavior due to RH/transient ischemia, as well as direct participation of EF (e.g., after administration of L-NMMA or a NO donor). Finally, clinical value of hyperemic response of SI, as well as applicability of this methodology, needs to be tested in population with cardiovascular risk factors and/or changes in radial pressure waveform (e.g., aged, diabetes mellitus, hypertension, smokers, and preeclampsia).

## Figures and Tables

**Table 1:** Characteristics of the subjects.

Variable	Value
Number	14
Sex, men/women	7/7
Age (years)	20.5 ± 0.5
Brachial systolic pressure (mmHg)	122 ± 9
Brachial diastolic pressure (mmHg)	70 ± 6
Heart rate (beats/min)	78 ± 8
Body mass index (kg/m^2^)	21.4 ± 2.6
Height (cm)	169 ± 11
Weight (kg)	61 ± 13
Total cholesterol (mg/dL)	165.9 ± 30.4
Low-density lipoprotein cholesterol (mg/dL)	89.9 ± 20.5
High-density lipoprotein cholesterol (mg/dL)	59.8 ± 15.4
Triglycerides (mg/dL)	80.2 ± 28.4
Fasting glucose (mg/dL)	82.3 ± 5.6
Smokers	None

Values expressed as mean ± standard deviation.

**Table 2:** Time delays of first and second peak of radial pulse pressure.

Variable	*t*_*S*_ (sec.)	*t*_*D*_ (sec.)	Δ*t*_*S*-*D*_ (sec.)
Baseline	0.11 ± 0.01	0.42 ± 0.02	0.31 ± 0.01
After ischemia at 60 sec.	0.12 ± 0.01	0.44 ± 0.02	0.32 ± 0.01
% of change	3.2	4.2	4.6
*P* value	0.002	<0.0001	<0.0001

Values expressed as mean ± standard deviation.
